# Comparison of *Escherichia coli* ST131 Pulsotypes, by Epidemiologic Traits, 1967–2009

**DOI:** 10.3201/eid1804.111627

**Published:** 2012-04

**Authors:** James R. Johnson, Marie-Hélène Nicolas-Chanoine, Chitrita DebRoy, Mariana Castanheira, Ari Robicsek, Glen Hansen, Scott Weissman, Carl Urban, Joanne Platell, Darren Trott, George Zhanel, Connie Clabots, Brian D. Johnston, Michael A. Kuskowski, the MASTER Investigators

**Affiliations:** Veterans Affairs Medical Center, Minneapolis, Minnesota, USA (J.R. Johnson, C. Clabots, B.D. Johnston, M.A. Kuskowski);; University of Minnesota, Minneapolis (J.R. Johnson, B.D. Johnston, M.A. Kuskowski);; Hôpital Beaujon, Clichy, France (M.-H. Nicolas-Chanoine);; The Pennsylvania State University, College Park, Pennsylvania, USA (C. DebRoy);; JMI Laboratories, North Liberty, Iowa, USA (M. Castanheira);; NorthShore University HealthSystem, Evanston, Illinois, USA (A. Robicsek);; Hennepin County Medical Center, Minneapolis (G. Hansen);; University of Washington, Seattle, Washington, USA (S. Weissman);; New York Hospital Queens, Flushing, New York, USA (C. Urban);; New York University School of Medicine, New York, New York, USA (C. Urban);; University of Queensland, Brisbane, Queensland, Australia (J. Platell);; University of Adelaide, Adelaide, South Australia, Australia (D. Trott);; University of Manitoba, Winnipeg, Manitoba, Canada (G. Zhanel)

**Keywords:** *Escherichia coli* infections, ST131, sequence type 131, CTX-M-15, fluoroquinolone resistance, antimicrobial resistance, extended-spectrum beta-lactamases, pulsed-field gel electrophoresis, bacteria, sequence typing

## Abstract

*Escherichia coli* sequence type 131 (ST131), an emerging disseminated public health threat, causes multidrug-resistant extraintestinal infections. Among 579 diverse *E. coli* ST131 isolates from 1967–2009, we compared pulsotypes (>94% similar *Xba*I pulsed-field gel electrophoresis profiles) by collection year, geographic origin, source, and antimicrobial drug–resistance traits. Of 170 pulsotypes, 65 had >2 isolates and accounted for 85% of isolates. Although extensively dispersed geographically, pulsotypes were significantly source specific (e.g., had little commonality between humans vs. foods and food animals). The most prevalent pulsotypes were associated with recent isolation, humans, and antimicrobial drug resistance. Predominant pulsotype 968 was associated specifically with fluoroquinolone resistance but not with extended-spectrum β-lactamase production or *bla*_CTX-M-15_. Thus, several highly successful antimicrobial drug–resistant lineages within *E. coli* ST131 have recently emerged and diffused extensively among locales while maintaining a comparatively restricted host/source range. Identification of factors contributing to this behavior of ST131 could help protect public health.

## Abstract 2

Certain high-prevalence pulsed-field gel electrophoresis types exhibited distinctive temporal patterns and epidemiologic associations.

The prevalence of resistance to fluoroquinolones and extended-spectrum cephalosporins in *Escherichia coli* has increased dramatically over the past decade. This increase is largely the result of the widespread emergence of a single disseminated *E. coli* clonal group, designated sequence type (ST) 131 according to multilocus sequence typing (MLST) (*1**,**2*). *E. coli* ST131 is characterized by serotype O25b:H4 and often produces CTX-M-15 or other extended-spectrum β-lactamases (ESBLs) (*3**–**5*). Unlike most other antimicrobial drug–resistant *E. coli*, ST131 derives from virulence-associated phylogenetic group B2 and typically exhibits multiple virulence factors, including adhesins, siderophores, toxins, and group 2 capsule (*1**–**7*). It thereby poses the dual threat of extensive antimicrobial drug resistance plus virulence.

By definition, ST131 is homogeneous with respect to housekeeping gene sequence across the 7 MLST loci; however, within-lineage genetic variation has been noted since ST131 was first described (*3**–**5*). Specifically, diversity of pulsed-field gel electrophoresis (PFGE) profiles has provided insights into the ecology of ST131. For example, the presence of ST131 isolates with similar PFGE profiles in widely dispersed locales and of isolates with quite different profiles in the same locale has suggested rapid and ongoing global dissemination of ST131 (*3**,**8*). Likewise, recovery of ST131 isolates with similar PFGE profiles from multiple household members (*9**–**12*) and from food animals (or retail meats) and humans (*13*) has suggested host-to-host or foodborne transmission, respectively, as potential mechanisms for dissemination of ST131.

However, relevant studies to date have included relatively few isolates, locales, and sources and limited time periods (*2**,**6*). In addition, the idiosyncratic nature of PFGE analysis precludes across-study comparisons. Thus, we analyzed 579 ST131 isolates from diverse sources according to a standardized PFGE protocol and then compared PFGE profiles with other characteristics, including geographic origin, time of collection, ecologic source, and antimicrobial drug–resistance traits.

## Materials and Methods

### Isolates

The 579 ST131 study isolates, some previously published (*3**,**9**–**12**,**14**–**19*), were compiled as a series of convenience samples from collaborators in diverse locales. The isolates came already identified as ST131 or as generic *E. coli* in need of screening for ST131 status. They derived mostly from collections assembled by investigators or reference laboratories on the basis of specific resistance phenotypes, O antigens, geographic origins, and/or clinical syndromes of interest. Some isolates were from cases or case series involving infected humans or animals with distinctive signs and symptoms and/or predisposing conditions (*9**–**12**,**14**,**15*).

Isolates were accompanied by data regarding date of isolation (or receipt in the reference laboratory), ecologic source (i.e., host species, food, or water), and locale of origin. For some isolates, data were available regarding resistance-associated characteristics, i.e., fluoroquinolone resistance; ESBL production; and presence of *bla*_CTX-M-15_, which encodes the CTX-M-15 ESBL variant. If not provided, this information was newly generated.

### ST131 Status

Of the 579 study isolates, 34 (5.9%) came already defined as ST131 by 7-locus MLST (http://mlst.ucc.ie/mlst/dbs/Ecoli). The remaining 545 (94.1%) were presumptively identified as ST131 in the study laboratory by PCR-based screening for ST131-specific single-nucleotide polymorphisms (SNPs) in *gyrB* and *mdh* (*17*). Full MLST was done de novo for 57 (10.5%) of these presumptive ST131 isolates, which represented diverse time periods, locales, sources, and resistance characteristics; in each instance, presumptive ST131 status was confirmed (SNP PCR specificity 100%; 95% CI 94%–100%). Thus, 91 (16%) isolates were directly confirmed by MLST to be ST131. Another 301 (52%) isolates represented pulsotypes with >1 MLST-confirmed ST131 isolate, which indirectly confirmed their ST131 status. Therefore, in total, 392 (68%) isolates were directly or indirectly confirmed by MLST to be ST131. Of the 31 earliest isolates (1967–1997), including the 5 earliest isolates (1967–1986), 32% were directly confirmed by MLST to be ST131.

### PFGE Analysis

PFGE analysis of *Xba*I-restricted total DNA of isolates was performed according to a standardized protocol (*20*) by a single observer in 1 laboratory. Profiles were captured and analyzed digitally by using BioNumerics software version 6.6 (Applied Maths, Austin, TX, USA). Marker lanes in each gel (*E. coli* O157:H7 strain g5244) enabled normalization within and across gels. Band positions were assigned manually, with computer assistance. The band tolerance setting, as derived empirically from analysis of multiple same-isolate profiles, was 1.15%.

Pairwise Dice similarity coefficients were used to define pulsotypes. Isolates exhibiting >94% profile similarity (≈3-band difference) to the index isolate for an established pulsotype, implying genetic similarity (*21*), were assigned to that pulsotype; others became the index isolate for a new pulsotype. Newly encountered pulsotypes were numbered sequentially. A PFGE profile dendrogram was constructed according to the unweighted pair group method for 87 (15%) of the isolates (selected randomly after inclusion of 2 representatives of each pulsotype with >6 members) plus the earliest isolated (1967) and earliest published (1985) isolates (*8*).

### Susceptibility Testing

Disk diffusion testing for ciprofloxacin susceptibility and ESBL production was performed on isolates of unknown fluoroquinolone or ESBL phenotype as described (*22**,**23*). Fluoroquinolone resistance was defined as nonsusceptibility to ciprofloxacin.

### Statistical Analyses

Geographic origin was categorized as United States (with 4 subregions—West, Midwest, South, and Northeast—as defined by the US Census Bureau [www.census.gov/geo/www/us_regdiv.pdf]), Canada, and other international locales combined. Ecologic source was categorized as human, companion animal, food animal, other animal, food, and water. Year of isolation/submission was assessed both continuously and categorically (e.g., pre-1990 vs. later).

Comparisons of proportions were tested by using 2-tailed Fisher exact (unpaired comparisons) and McNemar (paired comparisons) tests. Comparisons involving continuous variables were tested by using the Mann-Whitney U test (2-tailed). Other variables were assessed as independent predictors of selected pulsotype categories by using multivariable logistic regression analysis. The significance criterion was p<0.05.

## Results

### Isolate Origins and Characteristics

The 579 ST131 study isolates were derived from humans (486 [84%]), animals (77 [13%]), environmental sources (15 [3%]), and an unknown source (n = 1). Animal uses included companion (22 [4%]), food (45 [8%]), and other (10 [2%]). Environmental sources included food (6 [1%]) and water (9 [2%]). Geographic origins included the United States (446 [77%]), Canada (53 [9%]), and other international locales (80 [14.1%]). The US isolates were from the West (59 [10%]; 9 centers, 4 states), Midwest (180 [31%] centers, 11 states), South (98 [17%] centers, 9 states), and Northeast (109 [19%] centers, 7 states). Isolates from Canada were from 9 centers in 8 provinces. Other international isolates were from 11 centers in 11 countries (Australia, Chile, France, South Korea, Lebanon, India, Italy, Peru, Portugal, Spain, Switzerland). Dates of isolation/submission ranged from 1967 through 2009 (median year 2007). Twenty (4%) isolates were isolated/submitted during 1967–1989, the 5 earliest during 1967, 1982, 1983, 1985, and 1986; 22 (4%) were isolated/submitted during 1990–1999; and 537 (93%) were isolated/submitted during 2000–2009. Overall, 462 (80%) isolates were fluoroquinolone-resistant, 272 (47%) were ESBL-producers, and 188 (33%) had *bla*_CTX-M-15_.

### Pulsotypes

*Xba*I PFGE analysis resolved 170 distinct pulsotypes, each accounting for 1 isolate (105 pulsotypes) to 136 isolates (1 pulsotype, type 968). The 105 single-isolate pulsotypes collectively accounted for 105 isolates (18% of total); the 65 multiple-isolate pulsotypes accounted for the remaining 474 isolates (82% of total). Among the multiple-isolate pulsotypes, 12 contained >6 isolates each (i.e., >1% of the population), collectively accounting for 327 isolates (56% of total). The multiple-isolate pulsotypes contained 62 clusters of isolates (each comprising 2–5 isolates; total 150 isolates) with indistinguishable profiles.

### Temporal Patterns

Pulsotypes varied significantly by temporal occurrence. The 65 multiple-isolate pulsotypes and 12 high-prevalence pulsotypes were significantly associated with more recent dates of isolation/submission, relative to the low-prevalence and single-isolate pulsotypes (Table 1). Temporal variation was also evident among the 12 high-prevalence pulsotypes; 4 were significantly associated with later and 1 (type 955) with earlier occurrence (Table 1).

**Table 1:** Association of year of isolation/submission with pulsotype and other characteristics for 579 *Escherichia coli* sequence type isolates, 1967–2009*

Associated characteristic, specific trait†‡	Characteristic absent			Characteristic present		p value†
	No. isolates	Year, median (range)		No. isolates	Year, median (range)	
Pulsotype						
High-prevalence	252	2007 (1967–2009)		327	2007 (1987–2009)	<0.001
Multiple-isolate	105	2007 (1967–2009)		474	2007 (1982–2009)	<0.001
968	443	2007 (1967–2009)		136	2008 (1992–2009)	<0.001
812	547	2007 (1967–2009)		32	2007 (2005–2009)	0.03
987	568	2007 (1967–2009)		11	2008 (1998–2008)	0.004
955	572	2007 (1967–2009)		7	2002 (1993–2005)	0.001
1160	573	2007 (1967–2009)		6	2008 (2004–2009)	0.04
Resistance						
FQ	117	2003 (1967–2009)		462	2007 (2000–2009)	<0.001
ESBL	307	2007 (1967–2009)		272	2007 (2000–2009)	0.001
*bla*_CTX-M-15_	391	2007 (1967–2009)		188	2007 (2000–2009)	0.048
Source§						
Human	92	2003 (1982–2009)		486	2007 (1985–2009)	<0.001
Pet	556	2007 (1982–2009)		22	2008 (2002–2009)	<0.001
Food animal	533	2007 (1985–2009)		45	1997 (1982–2009)	<0.001
Food/water	563	2007 (1982–2009)		15	2003 (1993–2007)	<0.001
Region						
United States	133	2005 (1998–2009)		446	2007 (1967–2009)	0.007
West	520	2007 (1967–2009)		59	2007 (1982–2007)	<0.001
Midwest	399	2007 (1967–2009)		180	2008 (1986–2009)	<0.001
Canada	526	2007 (1967–2009)		53	2004 (1998–2004)	<0.001
International	499	2007 (1967–2009)		80	2008 (2002–2009)	<0.001

*ST, sequence type; FQ, fluoroquinolone; ESBL, extended-spectrum β-lactamase; *bla*_CTX-M-15_, gene encoding the CTX-M-15 ESBL; high-prevalence, pulsotypes with >6 isolates each; multiple-isolate, pulsotypes with >2 isolates each; international, non-US locales other than Canada. †Characteristics shown are those that yielded p<0.05 (Mann-Whitney U test, 2-tailed). ‡High-prevalence pulsotypes 800, 905, 1202, 807, 919, 595, and 797; other animal source; and origin from the US South or US Northeast did not exhibit a significant association with year. §Total for all source variables is 578 rather than 579 because source was unknown for 1 isolate.

Analysis of temporal prevalence trends (Figure) showed that the 12 high-prevalence pulsotypes accounted collectively for only 5% of 20 isolates during the earliest period (1967–1989) but for 58% of isolates during subsequent years (p<0.001). Three of these pulsotypes (988, 800, 812) were the top 1, 2, or 3 most prevalent, overall and within each interval from 1990 forward. These 3 types appeared sequentially by overall pulsotype prevalence (i.e., in 1990–1999 for type 968, in 2000–2002 for type 800, and in 2005 for type 812) and, except for type 800 in 2003, were detected continuously after first appearing. After it appeared, type 968 maintained a consistently high prevalence (>19%), whereas types 800 and 812 exhibited early prevalence spikes followed by sizeable drops. In contrast, the 9 other high-prevalence types appeared intermittently, which, depending on the pulsotype, was mostly in earlier years, later years, or sporadically throughout (Figure).

**Figure:**
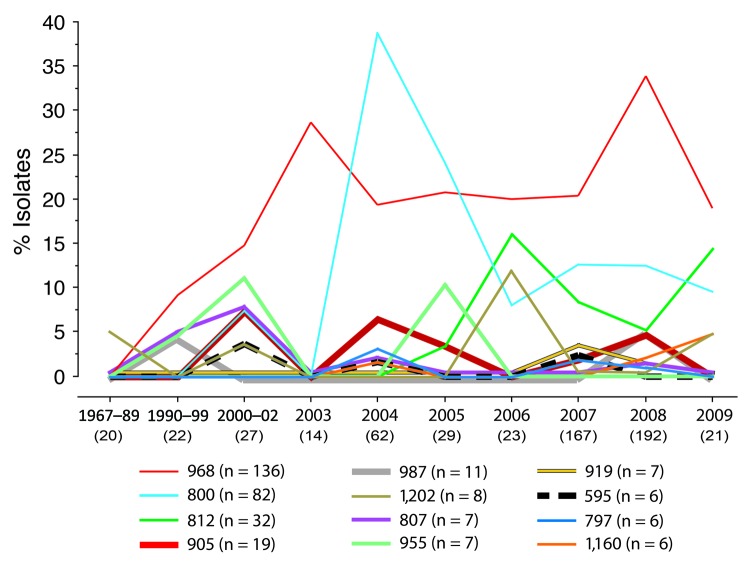
Prevalence over time of 12 high-prevalence *Xba*I pulsotypes among 579 *Escherichia coli* ST131 isolates. High-prevalence pulsotypes are those with >6 isolates (>1% of population) each. Years before 2003 are combined into 3 groups because of the small numbers of isolates. On the x-axis, the number of isolates for the particular period is shown in parentheses below the dates. y-axis prevalence values are based on the total number of isolates in the particular period.

**Figure A1:**
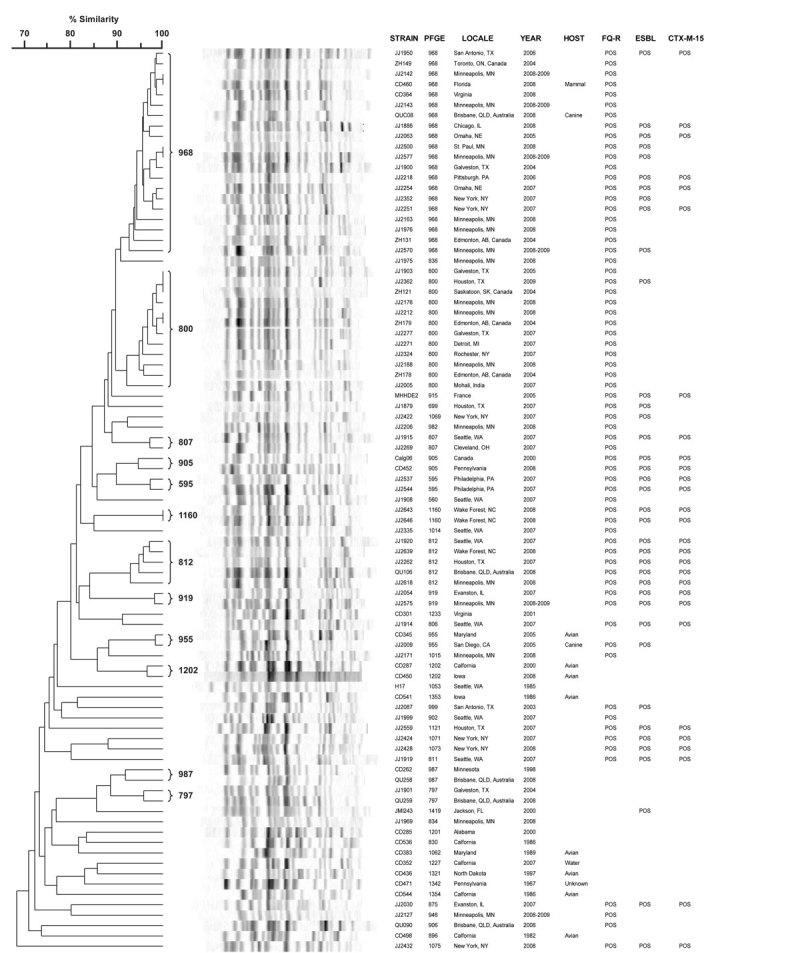
Pulsed-field gel electrophoresis profile dendrogram of 87 diverse isolates of *Escherichia coli* ST131. Isolates for the dendrogram, which represent a 15% subsample of the total population (n = 579), were selected randomly after deliberate inclusion of 2 representatives of each pulsotype with >6 members (indicated by brackets) plus the earliest previously reported isolate (from 1985) and the earliest present isolate (from 1967). YEAR, year of isolation or submission to reference laboratory; FQ-R, fluoroquinolone-resistant; ESBL, extended-spectrum β-lactamase; CTX-M-15, presence of *bla*_CTX-M-15_ (as detected by PCR); POS, positive (i.e., trait present); 2008–2009, interval during which isolate was collected. Scale is percent profile similarity.

A temporal trend was also evident in the PFGE dendrogram (Figure A1), which extended to 67% similarity. The more highly similar PFGE profiles in the upper region of the tree involved mostly recent isolates and higher prevalence pulsotypes, whereas the more basal, dissimilar profiles toward the lower region of the tree involved more older isolates (including isolates from 1967, 1982, 1985, and 1986) and low-prevalence pulsotypes.

### Geographic Distribution

The pulsotypes primarily exhibited a broad geographic distribution, yet there was some geographic segregation. Table 2 shows the number of mutually exclusive geographic regions (among 6 total) in which each of the 65 multiple-isolate pulsotypes were found. Only 18 of 65 multiple-isolate pulsotypes were limited to a single geographic region (p<0.001 for occurrence in 1 vs. multiple regions, McNemar test). Moreover, these 18 pulsotypes included only 2 (13 pulsotypes), 3 (4 pulsotypes), or 4 (1 pulsotype) isolates each and represented <50% of pulsotypes within their size category. In contrast, all pulsotypes including >5 isolates were found in multiple geographic regions, and 3 of the 6 pulsotypes comprising >8 isolates spanned all 6 geographic regions (Table 2). Table 3 shows the overlap among regions by the number of shared pulsotypes and by the number of isolates in these pulsotypes. Each region overlapped partially with every other region (Table 3).

**Table 2:** Geographic origin and ecologic source of 65 pulsotypes among 579 *Escherichia coli* ST131 isolates, 1967–2009*

No. isolates per pulsotype	No. pulsotypes	No. pulsotypes with isolate from indicated no. geographic regions†							No. pulsotypes with isolates from indicated no. ecologic sources‡			
		1	2	3	4	5	6		1	2	3	4
2	28	13	15	NA	NA	NA	NA		26	2	NA	NA
3	12	4	8	0	NA	NA	NA		10	2	0	NA
4	10	1	3	5	1	NA	NA		9	0	0	1
5	3	0	2	1	0	0	NA		1	2	0	0
6	3	0	0	0	2	1	0		2	1	0	0
7	3	0	0	0	1	2	0		2	0	1	0
>8	6	0	1	1	0	1	3		1	3	2	0

*ST, sequence type; NA, not applicable (no. regions or source groups exceeds no. isolates in pulsotype). †The 6 geographic regions included the US West, Midwest, South, and Northeast; Canada; and other international sites (collectively). ‡The 5 source groups included humans, companion animals, food animals, other animals, and food and water combined.

**Table 3:** Segregation, by geographic region, of multiple-isolate pulsotypes and of *Escherichia coli* ST131 isolates from the pulsotypes, 1967–2009*

Multiple-isolate pulsotype group				No. isolates, by geographic region, from pulsotype group (474)								No. pulsotypes, by geographic region, among isolate group (65)					
Source region	Total no. pulsotypes in group (65)	Total no. isolates in group (474)															
				US (358)	W (47)	MW (139)	S (84)	NE (88)	CAN (52)	INT (64)		US (61)	W (24)	MW (38)	S (23)	NE (31)	CAN (15)
US	61	463		NA	47	**139†**	84	88	50	***55‡***		NA					
West	24	348		**272†**	NA	105	62	58	41	***35‡***		24	NA				
MW	38	408		309	***33†***	NA	74	***63‡***	47	52		**38†**	15	NA			
South	23	350		272	***23‡***	108	NA	***57†***	43	***35‡***		23	10	17	NA		
NE	31	368		**287†**	***23‡***	106	68	NA	43	***38‡***		31	12	17	12	NA	
CAN	15	279		***201†***	***10‡***	**92†**	52	47	NA	***26***§		14	5	11	7	8	NA
INT	20	336		***231‡***	***17‡***	102	58	***54†***	41	NA		17	14	14	8	10	5

*Values in parentheses are n values. **Boldface** indicates significant associations; ***boldface italics*** indicate negative associations. Blank spaces indicate duplicated comparisons (mirror image of matrix). ST, sequence type; US, United States; W, West; MW, Midwest; S, South; NE, Northeast; CAN, Canada; INT, international (non-US locales other than Canada); NA, not applicable. †p<0.05 for indicated region vs. all other regions (Fisher exact test). ‡p<0.001 for indicated region vs. all other regions (Fisher exact test). §p<0.01 for indicated region vs. all other regions (Fisher exact test).

Against this background of broad geographic distribution, substantial geographic segregation of pulsotypes was evident. For example, at the isolate level, each region was negatively associated with at least 1 other region; the US West and non-Canadian international sites exhibited the greatest number of such negative associations, suggesting somewhat locale-specific pulsotype populations in these regions (Table 3). The only positive association between nonoverlapping regions involved the US Midwest and Canada.

Table 4 provides a pulsotype-level analysis of these geographic associations. For example, the high-prevalence and multiple-isolate pulsotypes were collectively significantly overrepresented in Canada, and the high-prevalence pulsotypes were also significantly underrepresented in the United States, specifically, in the US West. Among individual high-prevalence pulsotypes, 968 was overrepresented in the US Midwest and under-represented in the US West; 800 was over-represented in Canada and under-represented in the United States and the US West; 812 was over-represented in the US South; 987 was over-represented internationally (specifically in Australia, data not shown) and under-represented in the United States; and 1202 was over-represented in the US West (Table 4).

**Table 4:** Distribution, by geographic region, of *Escherichia coli* ST131 isolates from different pulsotype groups and from individual pulsotypes, 1967–2009*

Region	Total isolates, N = 579	High-prevalence pulsotypes, n = 327	Multiple-isolate pulsotypes, n = 474	Individual high-prevalence pulsotypes				
				968, n = 136	800, n = 82	812, n = 32	987, n = 11	1202, n = 8
US	446 (77)	**239 (73)†**	358 (80)	107 (79)	**55 (67)‡**	25 (78)	**1 (9)**§	8 (100)
West	59 (10)	**22 (7)†**	47 (10)	**4 (7)**§	**3 (4)‡**	5 (16)	0	**4 (50)†**
MW	180 (31)	56 (31)	139 (29)	**53 (39)‡**	24 (29)	7 (22)	1 (9)	2 (25)
South	98 (17)	64 (20)	84 (18)	26 (19)	17 (21)	**8 (25)†**	0	2 (25)
NE	109 (19)	53 (16)	88 (19)	24 (18)	11 (13)	5 (16)	0	0
CAN	53 (9)	**42 (13)**§	**52 (11)**§	14 (10)	**20 (24)**§	0	0	0
INT	80 (14)	46 (14)	64 (14)	15 (11)	7 (9)	7 (23)	**10 (91)**§	0

*Values are no (%) isolates. **Boldface** indicates p<0.05 in comparison with all other isolates (Fisher exact test). Pulsotypes are those among the 12 high-prevalence pulsotypes that exhibited a significant association with >1 geographic region. ST, sequence type; US, United States; MW, US Midwest; NE, US Northeast; CAN, Canada; INT, international (non-US locales other than Canada). †p<0.01 for indicated pulsotype group or pulsotype vs. all others (Fisher exact test). ‡ p<0.05 for indicated pulsotype group or pulsotype vs. all others (Fisher exact test). §p<0.001 for indicated pulsotype group or pulsotype vs. all others (Fisher exact test).

### Source Distribution

In contrast with the generally broad geographic distribution of pulsotypes, the source distribution was more restricted, and source-specific segregation predominated over across-source commonality. For example, only 13 of 65 multiple-isolate pulsotypes spanned multiple sources (p<0.001, McNemar test); most of these included only 2 sources each, and none included >4 (of 6 possible) sources (Table 2).

Likewise, the by-source distribution of pulsotypes (Table 5) showed less overall commonality than did geographic distribution. Still, it showed multiple positive and negative associations at the isolate and the pulsotype level. Specifically, isolates from humans were associated positively with pulsotypes comprising isolates from water and negatively with pulsotypes comprising isolates from companion animals, food animals, or food. Isolates from companion animals were associated positively with pulsotypes containing isolates from other animals, and isolates from food animals were associated positively with pulsotypes containing isolates from food (Table 5). In addition, pulsotypes containing isolates from food animals were associated negatively with pulsotypes containing isolates from humans, but they were associated positively with pulsotypes containing isolates from food (Table 5).

**Table 5:** Segregation, by ecologic source, of multiple-isolate pulsotypes and of *Escherichia coli* ST131 isolates from the pulsotypes, 1967–2009*

Ecologic source	No. pulsotypes, n = 65	Total no. isolates, n = 474	No. isolates							No. pulsotypes comprising isolates				
			HU, n = 412	CA, n = 21	FA, n = 28	OA, n = 7	Food, n = 4	Water, n = 2		HU, n = 57	CA, n = 8	FA, n = 13	OA, n = 2	Food, n = 2
HU	57	453	NA	19	***12†***	7	***1†***	2		NA				
CA	8	216	***178‡***	NA	8	**6**§	3	0		7	NA			
FA	13	43	***7†***	3	NA	1	**4†**	0		***5†***	2	NA.		
OA	2	140	118	**13‡**	**1†**	NA	1	0		2	1	1	NA	
Food	2	11	***1†***	2	**3**§	1	NA	0		1	1	**2**§	1	NA
Water	2	84	**82†**	***0***§	***0‡***	0	0	NA		2	0	0	0	0

*Blank spaces indicate duplicated comparisons (mirror image of matrix). **Boldface** indicates significant associations; ***boldface italics*** indicate negative associations. ST, sequence type; HU, human; CA, companion animal; FA, food animal; OA, other animal; NA, not applicable.  †p<0.001 for indicated source vs. all other sources (Fisher exact test). ‡p<0.01 for indicated source vs. all other sources (Fisher exact test). §p<0.05 for indicated source vs. all other sources (Fisher exact test).

Significant by-source segregation also was evident for individual pulsotypes (Table 6). Collectively, the multiple-isolate and high-prevalence pulsotypes were associated positively with humans and negatively with food animals and environmental sources, and high-prevalence pulsotypes were associated with companion animals. Furthermore, 5 high-prevalence pulsotypes were individually significantly distributed by source: type 968 was associated positively with pets and other animals and negatively with food animals and environmental sources, 800 was associated positively with humans and negatively with food animals, 812 was associated positively with humans, 1202 was associated negatively with humans and positively with food animals, and 955 was associated negatively with humans and positively with pets and environmental sources (Table 6). Five additional pulsotypes, comprising 2 food animal isolates each, were significantly associated with food animals (p = 0.004 for each; data not shown).

**Table 6:** Distribution, by ecologic source, of *Escherichia coli* ST131 isolates among different pulsotype groups and individual pulsotypes, 1967–2009*

Ecologic source	No. (%) isolates							
	Total isolates, N = 579	High-prevalence pulsotypes, n = 327	Multiple-isolate pulsotypes, n = 474	Individual high-prevalence pulsotypes				
				968, n = 136	800, n = 82	812, n = 32	1202, n = 8	955, n = 7
Human	486 (84)	**290 (89)†**	**412 (87)†**	117 (86)	**81 (99)†**	**31 (97)‡**	**1 (13)†**	0
Comp. animal	22 (4)	**19 (6)#**	21 (4)	**13 (10)†**	0	1 (3)	1 (8)	**2 (29)**§
Food animal	45 (8)	**8 (2)†**	**28 (6)†**	**0 †**	**0 †**	0	**6 (75)†**	2 (29)
Other animal	10 (1.7)	6 (2)	7 (1.5)	**6 (4)**§	0	0	0	0
Food/water	15 (2.6)	**4 (1)‡**	**6 (1)†**	**0 ‡**	1 (1)	0	0	3 (43)
Food	6 (1.0)	3 (0.9)	4 (0.8)	0	0	0	0	**3 (43)†**
Water	9 (1.6)	**1 (0.3)**§	**2 (0.4)†**	0	1 (1)	0	0	0

*Boldface indicates p<0.05 in comparison with all other isolates. Pulsotypes are those, among the 12 high-prevalence pulsotypes, that exhibited a significant association with >1 source group. ST, sequence type; comp., companion.  †p<0.001 for indicated pulsotype group or pulsotype vs. all others (Fisher exact test). ‡p<0.05 for indicated pulsotype group or pulsotype vs. all others (Fisher exact test). §p<0.01 for indicated pulsotype group or pulsotype vs. all others (Fisher exact test).

### Antimicrobial Drug Resistance

Fluoroquinolone resistance, ESBL production, and *bla*_CTX-M-15_ also segregated significantly by pulsotype in varied patterns (Table 7). The high-prevalence and multiple-isolate pulsotypes collectively and type 968 were associated positively with fluoroquinolone resistance but indifferently with ESBL production and *bla*_CTX-M-15_. In contrast, type 800 was associated positively with fluoroquinolone resistance but negatively with ESBL production and *bla*_CTX-M-15_, whereas types 905, 812, and 919 were associated positively with all 3 traits, and type 987 was associated negatively with all 3 traits (Table 7).

**Table 7:** Distribution, by antimicrobial drug resistance trait, of *Escherichia coli* ST131 isolates among different pulsotype groups and individual pulsotypes, 1967–2009*

Antimicrobial drug resistance trait	No. (%) isolates											
	Total no. isolates, N = 579	High-prevalence pulsotypes, n = 327	Multiple-isolate pulsotypes, n = 474	Individual high-prevalence pulsotypes								
				968, n = 136	800, n = 82	812, n = 32	905, n = 19	987, n = 11	1202, n = 8	919, n = 7	955, n = 7	797, n = 6
FQ-R	462 (80)	**293** (**90**)†	**401** (**85)**†	**133 (98)†**	**81 (99)†**	**32 (100)†**	**19 (100)‡**	**0†**	**1 (13)†**	**7 (100)**§	**1 (14)†**	**0 †**
ESBL	272 (47)	144 (44)	219 (46)	62 (46)	**12 (15)†**	**30 (94)†**	**17 (90)†**	**0†**	3 (38)	**7 (100)**§	**2 (29)**§	**0 ‡**
*bla*_CTX-M-15_	188 (33)	102 (31)	156 (33)	38 (28)	**5 (6)†**	**28 (88)†**	**16 (84)†**	**0‡**	**0 ‡**	**6 (86)**§	0	0

*Boldface indicates p <0.05 in comparison with all other isolates. Pulsotypes shown are those, among the 12 high-prevalence pulsotypes, that exhibited a significant association with >1 resistance trait. ST, sequence type; FQ-R, fluoroquinolone resistance; ESBL, extended-spectrum β-lactamase production; *bla*_CTX-M-15_, gene encoding CTX-M-15. †p<0.001 for indicated pulsotype group or pulsotype vs. all others (Fisher exact test). ‡p<0.05 for indicated pulsotype group or pulsotype vs. all others (Fisher exact test). §p<0.01 for indicated pulsotype group or pulsotype vs. all others (Fisher exact test).

### Multivariable Analysis

All 3 resistance traits, plus several source groups and geographic regions, exhibited significant associations with year of isolation/submission (Table 1), suggesting possible confounding by temporal correlations among variables. Thus, we used multivariable logistic regression analysis to assess for independent associations of selected predictor variables with pulsotype. Separate models were constructed for the 3 most prevalent pulsotypes, the high-frequency pulsotypes, and the multiple-isolate pulsotypes, by using as candidate predictor variables 1 representative from each epidemiologic or resistance category (year, ecologic source, locale, fluoroquinolone phenotype, and ESBL status). ESBL status was a significant predictor in all 5 resulting models, as was fluoroquinolone resistance in 4 models (fluoroquinolone resistance was excluded from the fifth model because of its 100% prevalence in pulsotype 812), year in 3 models, and human source in 2 models (Table 8). In contrast, US origin (the representative geographic variable) was not a significant predictor in any model.

**Table 8:** Results of multivariable logistic regression analysis for predictors of selected pulsotype categories among 579 *Escherichia coli* ST131 isolates, 1967–2009*

Outcome variable, significant predictor variables†	Odds ratio (95% CI)	p value	Nagelkerke R^2^ for model‡
Pulsotype 968			0.18
Human source	0.20 (0.09–0.46)	<0.001	
FQ-R	55.04 (10.63–285.03)	<0.001	
ESBL production	0.63 (0.41–0.90)	0.03	
Pulsotype 800			0.31
Human source	10.61 (1.32–85.48)	0.03	
FQ-R	46.50 (3.75–576.74)	.003	
ESBL production	0.10 (0.05–0.19)	<0.001	
Pulsotype 812			0.21
Year of isolation/submission	1.47 (1.07–2.03)	0.02	
ESBL production	17.53 (4.02–76.07)	<0.001	
High-prevalence pulsotypes			0.16
Year of isolation/submission	1.09 (1.03–1.55)	0.003	
FQ-R	4.04 (2.22–7.34)	<0.001	
ESBL production	0.47 (0.32–0.70)	<0.001	
Multiple-isolate pulsotypes			0.13
Year of isolation/submission	1.07 (1.02–1.13)	0.009	
FQ-R	3.42 (1.76–6.66)	<0.001	
ESBL production	0.42 (0.24–0.71)	0.001	

*ST, sequence type; FQ-R, fluoroquinolone resistance/resistant; ESBL, extended-spectrum β-lactamase; high-prevalence pulsotypes, 12 pulsotypes that contained >6 isolates (>1% of population) each; multiple-isolate pulsotypes, 65 pulsotypes that contained >1 isolate each. †Variables shown are those that yielded p values of <0.05. With 1 exception, each model included the following candidate predictor variables: human source (vs. other sources), US origin (vs. Canada or other international locale origin), year of isolation/submission, FQ-R, and ESBL production. All pulsotype 812 isolates were FQ-R; thus, FQ-R could not be included in that model. ‡Nagelkerke R^2^ provides an estimate of the total amount of variance accounted for by the model, i.e., the model's explanatory power. Values range from 0 (no explanatory power) to 1.0 (complete prediction).

### Indistinguishable PFGE Profile Isolates

We also assessed associations with other variables for the 7 largest clusters of isolates with indistinguishable PFGE profiles; each cluster contained 4–5 isolates. Of the 31 constituent isolates, 28 were recent (2007–2009) and the other 3 were from 2002 or 2004. Of the 7 clusters, 6 included isolates from multiple locales, from multiple continents in 3 instances. In contrast, only 3 clusters came from multiple host species. Whereas each cluster was internally homogeneous for fluoroquinolone phenotype (6 all-resistant clusters, 1 all-susceptible cluster), 4 were internally heterogeneous according to ESBL and/or *bla*CTX-M-15 status.

## Discussion

We used PFGE analysis to define population structure among 579 diverse *E. coli* ST131 isolates and then assessed temporal, geographic, ecologic, and resistance trait associations for the various pulsotypes, i.e., presumed sub-ST genetic lineages. Our findings support 4 main conclusions. First, although ST131 is highly diverse at the pulsotype level, a small number of high-frequency pulsotypes predominate, and pulsotype 968 accounts for 24% of the population. Second, pulsotypes differ in prevalence over time; high-prevalence pulsotypes tend to occur in more recent years, consistent with recent emergence and expansion, implying greater fitness. Third, whereas broad geographic distribution predominates over locale-specific segregation, implying widespread dispersal rather than localized endemicity, segregation by ecologic source predominates over across-source commonality, implying niche adaptation rather than broad host-range capability and interspecies transmission. Fourth, resistance traits (i.e., fluoroquinolone resistance, ESBL production, and *bla*_CTX-M-15_) are highly pulsotype-specific, suggesting predominantly subclonal distribution.

The striking prevalence disparities among pulsotypes suggest that certain pulsotypes, especially the exceptionally successful pulsotype 968, possess fitness advantages over others. In retrospect, pulsotype 968 accounted for all previously reported household clusters, 3 of which involved serious or fatal disease in >1 household members (*9**–**12*). A possible founder effect for type 968 is unlikely because the pulsotypes that were detected earliest were mostly low-prevalence types; higher prevalence pulsotypes appeared only later, seemingly outcompeting the low-prevalence types. In regard to possible fitness advantages, ESBL production and *bla*_CTX-M-15_ were significantly associated with several high-prevalence pulsotypes and were present in most or all of their members (Table 7). However, they were not significantly associated with (predominant) pulsotype 968 and so are unlikely the main explanation for the recent expansion of ST131, of which pulsotype 968 was the single main component (Figure). In contrast, fluoroquinolone resistance was significantly associated with each of the 4 most prevalent pulsotypes and collectively with the 12 high-prevalence and 65 multiple-isolate pulsotypes. Thus, fluoroquinolone resistance may have made a major contribution to the recent expansion of ST131.

Although the predominant pattern was broad dispersal of pulsotypes, localized segregation also occurred. These trends imply considerable ongoing dissemination and intermixing of ST131 lineages among locales (sufficient to largely preclude establishment of locale-specific populations) but with variable degrees of intermixing versus segregation by locale and pulsotype. For example, the US Midwest and Canada shared pulsotypes more extensively than did other regions. Conversely, non-Canadian international locales and the US West had less pulsotype commonality with other regions (i.e., had more highly locale-specific populations) than did other locales. Several high-prevalence pulsotypes similarly exhibited distinct patterns of distribution and were variably concentrated in specific regions. Similar patterns have been described previously for ST131 but in lesser detail and without statistical analysis (*3**,**6**,**8**,**17**,**24**,**25*). The undefined mechanisms for the ongoing dispersal of ST131, possibly including international travel and commerce, wild bird migration, and foodborne or waterborne transmission, and its limited locale-specific segregation by pulsotype warrant study.

The associations of specific pulsotypes with different ecologic sources are relevant to the dispersal mechanisms of ST131. In contrast to the striking geographic dissemination of pulsotypes, we also found some evidence of niche segregation. Positive associations for niche segregation were found between humans and water, companion animals and other animals, and food animals and food. In contrast, negative associations were found between humans and most other sources, companion animals or other animals and food or food animals, and water and companion animals or food animals. These findings implicate humans as the source for ST131 isolates found in water and implicate food animals as the source for isolates found in food. In contrast, and consistent with findings in most, but not all, previous studies (*13**,**26**–**28*), these findings indicate that food animals and food are not major sources of ST131 for humans. They also suggest no special pet­–human commonality of ST131 pulsotypes, notwithstanding some well-documented overlap (*29*). This argues against pets and the food supply as major vehicles for dissemination of ST131 strains among humans. Indeed, in 1 study, 7% of healthy humans were found to be colonized intestinally with an ST131 strain (*30*); thus, humans may be the main reservoir for human-associated strains. A larger, more current, and more systematically assembled study population is needed to confirm the findings of the present study.

Until now, the earliest reported isolate of ST131 was from a patient with urosepsis in 1985 (*8*). Here, we report 3 earlier isolations, from 1967, 1982, and 1983, none of which were from a high-prevalence pulsotype. This finding documents the presence of ST131 decades before its emergence as a disseminated human pathogen and suggests an opportunity to compare early isolates with recent isolates for characteristics that might confer enhanced fitness, possibly contributing to the emergence of ST131.

Our study had limitations. First, the population was a convenience sample with multiple possible sources of bias. Second, despite considerable diversity, the population was not balanced; it predominantly comprised recent isolates from human in the United States, reducing both generalizability and power for comparisons involving other times, sources, and regions. Third, minimal associated data (especially clinical details) were available for many isolates, limiting the possible epidemiologic analyses. Fourth, PFGE profiles reflect genetic relationships only indirectly and require subjective interpretation. Fifth, the multiple comparisons could have produced spurious associations by chance alone. However, the proportion of comparisons yielding a significant p value was much greater, and the associated p values much smaller, than should occur by chance alone. Last, the 94% PFGE similarity pulsotype criterion was somewhat arbitrary and possibly suboptimal; however, an alternate 100% similarity criterion yielded qualitatively similar conclusions.

Our study also had strengths. The population was the largest reported to date for ST131 (*2*) and the most extensively distributed by time, source, and region. The PFGE analysis was conducted by 1 experienced observer in 1 laboratory by using software that enabled concurrent comparisons for all isolates. Diverse univariable and multivariable statistical approaches were used, pulsotypes were analyzed collectively and individually, and PFGE profiles were assessed by using 2 similarity thresholds (94% and 100%) and in a dendrogram.

Thus, within a large, diverse collection of *E. coli* ST131 isolates, we documented extensive PFGE profile diversity and a predominance of certain high-prevalence pulsotypes (particularly pulsotype 968, 24% overall) that exhibited distinctive temporal patterns of emergence. Notwithstanding some geographic localization, pulsotypes were extensively dispersed by region. In contrast, they were more highly source specific; in particular, isolates from humans exhibited almost no commonality with isolates from food animals or foods. Pulsotype 968 was much more closely associated with fluoroquinolone resistance than with ESBL production or *bla*_CTX-M-15_, suggesting a greater role for fluoroquinolone resistance than ESBLs in the expansion of this dominant pulsotype and ST131 in general. These findings considerably advance our understanding of the genetic structure, ecology, geographic distribution, and emergence of this widely disseminated antimicrobial drug–resistant pathogen, which represents a growing public health threat.
